# Stress Response and Virulence of Heat-Stressed *Campylobacter jejuni*

**DOI:** 10.1264/jsme2.ME14020

**Published:** 2014-10-02

**Authors:** Anja Klančnik, Darinka Vučković, Polona Jamnik, Maja Abram, Sonja Smole Možina

**Affiliations:** 1 Department of Food Science and Technology, Biotechnical Faculty, University of Ljubljana, Jamnikarjeva 101, SI-1111 Ljubljana, Slovenia; 2 Department of Microbiology, School of Medicine, University of Rijeka, Brace Branchetta 20, HR-51000 Rijeka, Croatia

**Keywords:** *Campylobacter jejuni*, heat-stress response, virulence, food safety

## Abstract

Thermotolerant *Campylobacter* spp. frequently cause bacterial gastroenteritis in humans commonly infected through the consumption of undercooked poultry meat. We examined *Campylobacter jejuni* heat-stress responses *in vitro* after exposure to 48°C and 55°C. The *in vivo* modulation of its pathogenicity was also investigated using BALB/c mice intravenously infected with stressed *C. jejuni*. Regardless of the bacterial growth phase, the culturability and viability of *C. jejuni in vitro* was reduced after exposure to 55°C. This correlated with the altered protein profile and decreased virulence properties observed *in vivo*. Heat stress at 48°C elicited the transition to more resistant bacterial forms, independent of morphological changes or the appearance of shorter spiral and coccoid cells. This treatment did not cause marked changes in bacterial virulence properties *in vivo*. These results indicated that the characteristics and pathogenicity of *C. jejuni* in response to heat stress are temperature dependent. Further studies on the responses of *C. jejuni* to stresses used during food processing, as well as the modulation of its virulence, are important for a better understanding of its contamination and infective cycle, and will, thus, contribute to improved safety in the food production chain.

*Campylobacter* spp. have become one of the most frequent agents of common zoonotic food-borne diseases (6). The handling and consumption of contaminated poultry meat products have been identified as the most common sources of human *Campylobacter* spp. infection; however, raw vegetables and environmental water sources are also potential reservoirs (11).

Although *Campylobacter* spp. are generally regarded as being sensitive to the different environmental conditions exterior to animals or humans, they appear to be more resistant to stress than previously thought (12, 18). Thus, effective approaches to reduce human illnesses associated with these food-borne pathogens are needed in the food production and supply chains. The evolved mechanisms that allow *Campylobacter* spp. to deal with environmental stress conditions, and even develop resistance or potentially cross-protection to different stresses, need to be more fully elucidated. In addition to complex interaction, any single stress response may be regulated at different cellular levels; therefore, the relationships among the ecology, survival characteristics, and virulence properties of *Campylobacter* spp. need to be examined in more detail (7, 22, 24, 36).

Heat-shock regulation in *C. jejuni* has been shown to play an essential role in colonization of the intestinal tract (20). *Campylobacter* spp. are exposed to different thermal treatments during food processing, distribution, and storage that should be sufficient to either inhibit or inactivate them (34). To avoid the possible heat resistance of *Campylobacter* spp. in food, which may be harmful to public health, it is important to clarify changes in their cellular physiology as well as the activation of other global regulators as a consequence of inappropriate thermal treatments.

A proteomic approach can describe dynamic protein composition variations in a cell that constantly adjusts to meet the challenges of environmental changes, and can also be used to investigate cell responses to various stress conditions relevant to food processing and food safety (29, 38). Microbial adaptation during stress challenges is crucial not just for pathogen survival outside of the host, but also during host–pathogen interactions, and, thus, for bacterial pathogenicity (18, 19). A mouse model has proved to be an invaluable tool for understanding the pathogenesis of *C. jejuni* as well as the factors involved in host defense mechanisms (19, 37). Intravenous challenges in mice may be used to compare the virulence of bacterial strains exposed to various stress conditions. Differences in dissemination capabilities and tissue invasion can indicate changes in bacterial virulence potential.

However, the physiology of *C. jejuni* exposed to heat and after heat-shock regulation is poorly understood. The synthesis of a group of highly conserved heat-shock proteins (Hsps) is induced by heat stress (23). These proteins are known to have various roles in cell physiology, ribosome stability, stringent responses, temperature sensing, and the control of ribosomal function (1, 5, 16, 39). Dasti *et al.* (4) confirmed the role of Hsps in the thermotolerance and growth of *C. jejuni* in the chicken intestine. *C. jejuni* can use more than one strategy to simultaneously regulate sets of heat-shock-expressed genes and respond to temperature changes or other stresses, thereby enhancing its survival in the environment (4, 35). Incubation outside the normal temperature range of growth is one of the environmental inducers of the viable but non-culturable (VBNC) form (26, 27).

In the present study, we examined the stress responses of *C. jejuni* from different growth phases after heat treatments at 48°C and 55°C. These temperatures were chosen to mimic the temperatures applied in poultry processing plants: 48°C for locations close to the hot water in the defeathering environment and 55°C for scalding (15). We also explored the ability of this bacterium to acquire heat resistance after being previously exposed to another stress *i.e.* starvation. Changes in bacterial viability, culturability, morphology, and its protein profile were investigated. We also examined the influence of the stress response of *C. jejuni* on its virulence potential using a previously established experimental model of systemic murine campylobacteriosis (37).

## Materials and Methods

### Bacterial growth and stress conditions

*C. jejuni* K49/4 was isolated from poultry meat, identified phenotypically, and subcultured prior to the experimental test conditions. Microaerobic growth (5% O_2_, 10% CO_2_, 85% N_2_) in Preston broth (Oxoid, Hampshire, UK) containing 5% (v/v) defibrinated horse blood (Oxoid) at 42°C for 9 h induced the entry of these cultures into the exponential growth phase, and for 15 h, into the stationary growth phase (1). To produce heat-shock stress, the bacterial cells harvested from each growth phase were exposed to temperature shifts from 42°C to 48±1°C or 55±1°C using thermoblock (Eppendorf Thermomixer comfort, Eppendorf AG, Hamburg, Germany) for 3, 10, 20, or 30 min, and these cells were then shortly cooled on ice to achieve 42°C before being analyzed. Untreated *C. jejuni* from both growth-phase cultures were used as controls and kept at 42°C throughout the experiment. To evaluate resistance to the heat treatments, 5 μg mL^−1^ chloramphenicol was added as an inhibitor of protein synthesis before 5 h of starvation and the subsequent heat stress. *C. jejuni* cells were harvested by centrifugation (12,000×*g* for 5 min at 4°C), washed, resuspended in Ringer’s solution supplemented with 5 mM KH_2_PO_4_ (Kemika, Zagreb, Croatia), and incubated microaerobically for 5 h at 42°C for pre-starvation.

### Culturability, viability, and morphology assays

Culturability was determined as colony-forming units per milliliter (CFU mL^−1^), and viability was determined using the LIVE/DEAD *Bac*Light system (L-7012; Molecular Probes, Eugene, OR, USA) under the Eclipse TE300 microscope (Nikon, Tokyo, Japan), as previously described (1, 18). Viability was given as the percentage of viable cells in relation to the total number of cells obtained, as determined on 20 randomly chosen microscopic fields per filter for each evaluated sample. The numbers of coccoid and spiral cells were also determined. All experiments were independently repeated three times. The morphology of *C. jejuni* was assessed using transmission electron microscopy (Philips CM 100, Philips Electronics N.V. Eindhoven, the Netherlands). Cells were prepared as reported previously (17).

### Protein extraction and two-dimensional gel electrophoresis

*C. jejuni* cells from the exponential growth phase exposed to 48°C or 55°C for 20 min were investigated in the two-dimensional (2-D) gel electrophoresis analysis. After the treatments, these cells were harvested by centrifugation (12,000×*g* for 5 min at 4°C), washed with Ringer’s solution, and resuspended in extraction solution (40 mM Tris-HCl, pH 7.5, 4% [w/v] 3-[(3-cholamidopropyl) dimethylammonio]-1-propanesulfonate [CHAPS], 65 mM dithiothreitol, and protease inhibitor cocktail (1 tablet 10 mL^−1^ buffer) (Complete, Mini; Roche). To extract the protein, the cells were disrupted using three cycles of vortexing with zirconia/silica beads (BioSpec Products; diameter, 0.5 mm) for 1 min each, with 1-min intervals of cooling on ice. The homogenate was centrifuged (20,000×*g* for 20 min at 4°C), and the protein concentration in the supernatant (extract) was measured by the method of Bradford (2). The extract was then purified using 2-D Clean Up kits (GE Healthcare, Sweden).

Two-dimensional (2-D) gel electrophoresis was performed according to Görg (9), with minor modifications. The protein samples (100 μg) were resuspended in rehydration solution (9 M urea, 2% [w/v] CHAPS, 2% [v/v] immobilized pH gradient [IPG] buffer, 18 mM dithiothreitol, 0.002% [w/v] bromophenol blue), and loaded onto 13-cm IPG strips (4–7, GE Healthcare). After rehydration, isoelectric focusing was carried out using Multiphor II (GE Healthcare). The proteins were focused at: 300 V (gradient over 1 min), 3500 V (gradient over 1.5 h), and 3500 V (fixed for 4.33 h). After isoelectric focusing, the IPG strips were equilibrated for 15 min in SDS equilibration buffer (50 mM Tris-HCl, pH 8.8, 6 M urea, 30% [v/v] glycerol, 2% [w/v] SDS, 0.0002% [w/v] bromophenol blue) containing 1% dithiothreitol, and for an additional 15 min in SDS equilibration buffer containing 4.8% iodoacetamide. The second dimension (SDS–PAGE) was performed on 12% SDS-polyacrylamide gels on an SE 600 vertical discontinuous electrophoresis system (Hoeffer Scientific Instruments). The 2-D gels were stained using fluorescent Sypro Ruby (Invitrogen), and then recorded using an Artixscan 1800f scanner (Microtek). The protein profiles between control and treated cells were then compared using the 2-D Dymension software, version 2.02 (Syngene). 2-D electrophoresis was run at least twice for each sample.

### *In vivo* experiments

Eight to twelve week old male BALB/c (H-2^d^) mice were used in all of the *in vivo* experiments. These mice were obtained from the Central Animal Facility of the School of Medicine, University of Rijeka, Croatia, and were given standard laboratory rodent food (Mucedola, Milan, Italy) and water *ad libitum*. Experiments were conducted in accordance with the guidelines of the International Guiding Principles for Biomedical Research Involving Animals (25). The Ethical Committee at the School of Medicine, University of Rijeka approved all of the animal experiments described here. Mice were infected intravenously via the lateral tail vein with a single dose (200 μL) of 0.5 to 1.0×10^9^ CFU mL^−1^ unstressed (control) or heat-stressed *C. jejuni* cells, as determined by the turbidity of the bacterial suspension and confirmed retrospectively by plating the inoculum on blood agar and incubation microaerobically for 48 h at 42°C. At one, three, and eight d post-infection, the mice were sacrificed and their livers and spleens were removed aseptically and dissected from the surrounding tissue, with the *C. jejuni* CFU in the livers and spleens determined as previously described (19, 37). In bacterial organ burden experiments, two sets of mice were infected with each of the heat-treated or control *C. jejuni*, with at least three mice per group. These experiments were repeated three times, and the data from all of the replicate experiments were pooled and presented as means±standard deviations.

### Statistical analysis

Differences in the bacterial counts among all of the experimental groups were calculated using the Kruskal–Wallis test, while the Mann–Whitney test was used to define differences between pairs of groups. All of the statistical values were considered significant at a *p* level <0.05. Statistical analyses were performed using SPSS 15.0 for Windows (Statsoft, Tulsa, OK, USA).

## Results

### Physiology and morphology of C. jejuni under heat stress

**Fig. 1:**
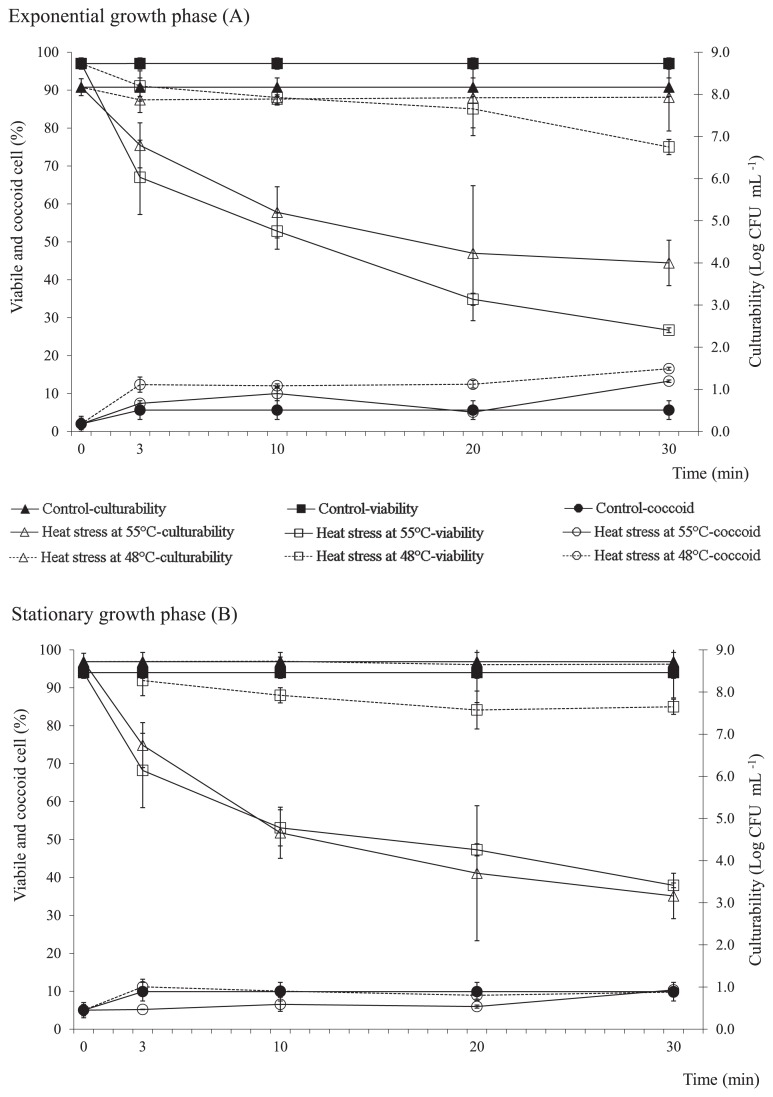
Viability, coccoid cell formation, and culturability in exponential (A) and stationary (B) growth phase *C. jejuni* exposed to 48°C or 55°C, as indicated. Data are expressed as means±standard deviations.

The viability of exponential growth phase cells exposed to 48°C was reduced by approximately 20% after 30 min, with the coccoid cell number increasing by 10% (Fig. 1A). This pattern was not observed in the stationary growth phase cultures, in which no significant differences were observed in culturability or coccoid cell numbers prior to and after the heat stress (Fig. 1B). The decline in viability in the stationary growth phase cultures was less pronounced than that in the exponential growth phase cultures (Fig. 1). Regardless of the growth phase, the heat treatment at 55°C, which lasted for over 3 min, resulted in a progressive decline in viability and culturability of the bacterial cells. Cell viability had already decreased by 50% after 10 min under the 55°C stress challenge, while culturability declined by at least 3.0 log units (Fig. 1). The proportion of coccoid cells was 20% (Fig. 1).

**Fig. 2:**
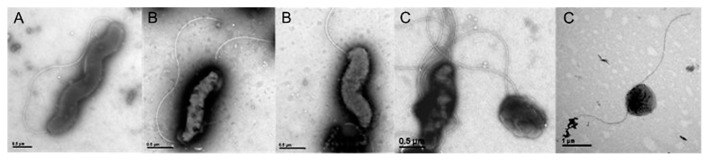
Representative electron microscopy showing control *C. jejuni* cells (A) and the effects of heat stress at 48°C (B) and 55°C (C) on bacterial morphology. Scale bars: as indicated.

Transmission electron microscopy was used to examine morphological changes in *C. jejuni* and representative electron microscopy images were presented after evaluating at least 10% of randomly chosen copper grids for each evaluated sample (Fig. 2). Morphological changes were more visible among cells exposed to 55°C, which significantly changed their morphology from that in the control culture (Fig. 2). The temperature shift provoked morphological changes in *C. jejuni* from spiral to predominant shorter spiral and coccoid forms, indicating a possible transformation to the VBNC form.

### Protein profile of *C. jejuni* under heat stress

**Fig. 3:**
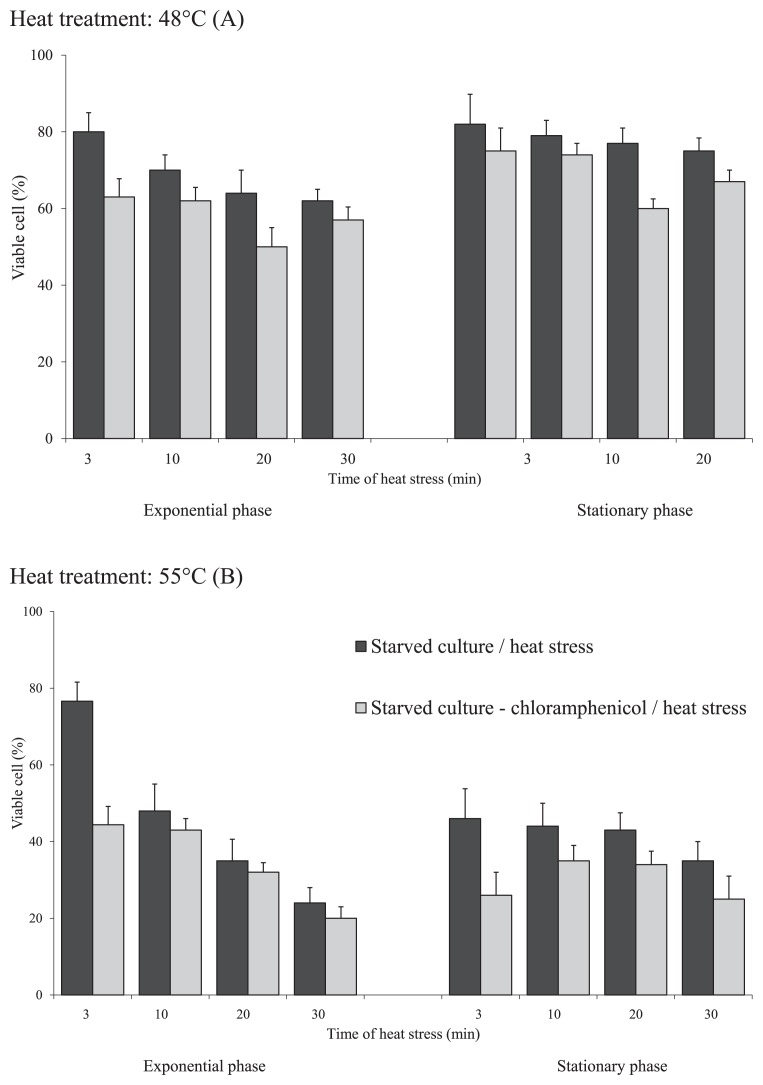
Resistance to heat stress, evaluated according to the viability of exponential and stationary growth phase *C. jejuni* without or with chloramphenicol added for 5 h before pre-starvation and subsequent exposure to the heat treatment at 48°C (A) or 55°C (B), as indicated. Data are expressed as means±standard deviations.

To determine the heat stress resistance of *C. jejuni* cells from both growth phases, we exposed the cultures to another stress, starvation, prior to the heat treatment. Acquired resistance to heat stress was more prominent in the exponential growth phase culture at 48°C (Fig. 3). When chloramphenicol was added before the exposure to 5 h starvation, the reduction observed in cell viability after heat stress at either 48°C or 55°C was more pronounced in treated cells than in non-treated cells, in which protein synthesis was maintained. We assumed that starvation induced protein synthesis in *C. jejuni* cells from the exponential growth phase, and may be important for the acquisition of heat resistance. Therefore, we further investigated the protein profile and virulence of *C. jejuni* under heat stress using cultures from the exponential growth phase.

**Fig. 4:**
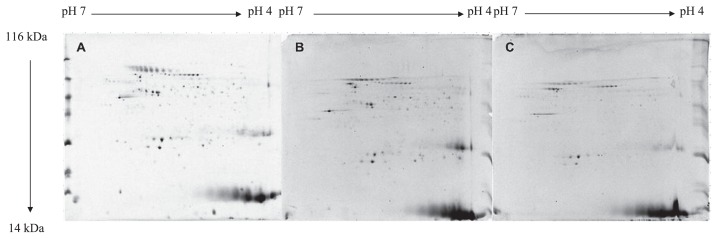
Representative protein profiles of the *C. jejuni* under the exponential growth phase, for the control cells (A) and the cells heat treated at 48°C (B) and 55°C (C), for 20 min.

We compared the protein profiles of the untreated (control) and heat-treated (48°C, 55°C, for 20 min) cultures. The reduced numbers of proteins detected in the gels corresponded to decreases in both viability and culturability. Following the heat treatment at 48°C for 20 min, the expression of 36 proteins was lower and that of 4 proteins was higher than that of the control cells, while 3 proteins were absent. The exposure of *C. jejuni* to 55°C generally reduced the number of proteins detected, with 29 down-regulated signals being observed. The expression of only one protein was higher than that of the corresponding control (Fig. 4).

### Virulence of *C. jejuni* after heat stress

To define the effects of the stress response to heat treatment on the virulence of *C. jejuni*, we assessed systemic bacterial infections in a murine model. Bacterial numbers were assessed in the livers and spleens of mice 1, 3, and 8 d after an intravenous infection with untreated (control) or heat-treated *C. jejuni* (48°C for 3 or 20 min, 55°C for 3 min).

**Fig. 5:**
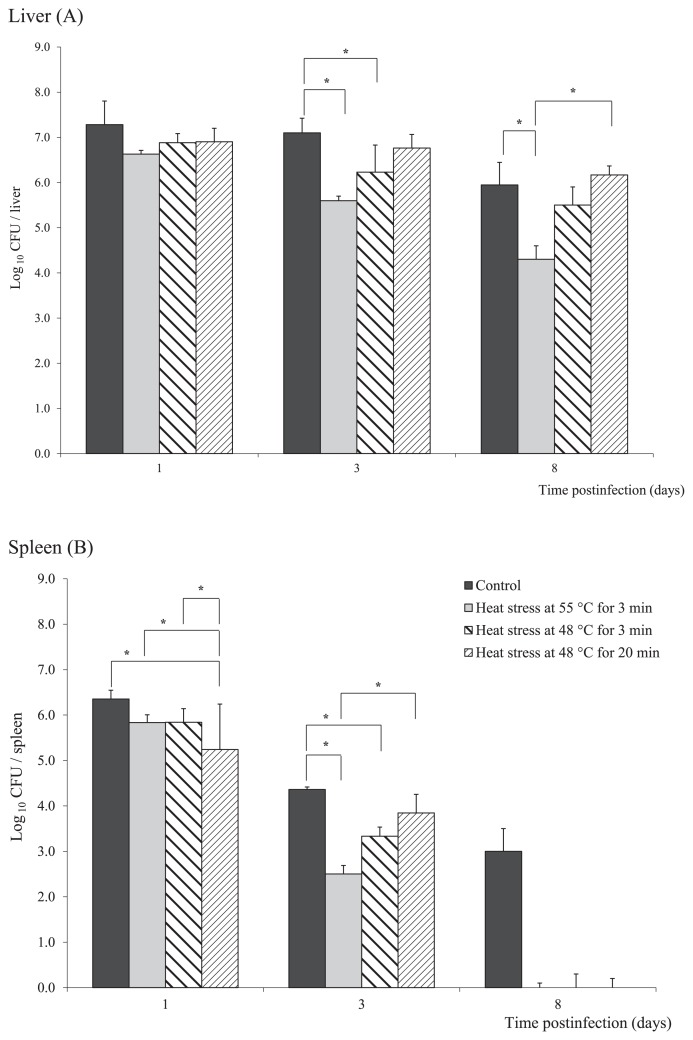
*C. jejuni* cell numbers in the liver (A) and spleen (B) of BALB/c mice intravenously infected with exponential growth phase *C. jejuni* cells, as untreated control cells, and cells exposed to heat treatments at 55°C for 3 min or 48°C for 3 min and 20 min, as indicated. Data are expressed as means±standard deviation of *C. jejuni* log_10_ CFU organ^−1^. (*p≤0.05).

As expected, control bacteria efficiently established systemic infections with a lower bacterial burden being detected in the mouse spleen than in the liver for the whole experimental period (Fig. 5). Heat-stressed *C. jejuni* also quickly disseminated to the spleen and liver, except those exposed to 55°C for 20 min, which were not capable of inducing a systemic infection.

The number of heat-stressed *C. jejuni* recovered from the livers and spleens was reduced 1 d post-infection (Fig. 5). The number of bacteria isolated from the livers 3 and 8 d post-infection was markedly reduced when bacteria treated with 55°C were used to infect the mice (a reduction of 2 log units after 8 d) (Fig. 5A). The same stresses caused marked decreases in bacterial numbers within the spleen, particularly 8 d post-infection, when heat-stressed *C. jejuni* cells could not be cultivated from the spleen regardless of the stress applied (Fig. 5B).

## Discussion

Despite its particular growth requirements, *C. jejuni* has developed mechanisms for survival in diverse environments, in which it is subjected to various stresses, including high temperatures. The results of the present study clearly demonstrated that exposure to 48°C and to 55°C for a short time (3 min) changed the morphology and protein profile of *C. jejuni*, but did not affect its infectivity. However, a longer exposure to 55°C (20 min) completely abolished the virulence of *C. jejuni*, and generally reduced the number of proteins detected. Such responses and the adaptation of this foodborne pathogen to heat stress applied during food processing may constitute a microbial hazard.

The exposure of *C. jejuni* to 48°C as well as 55°C induced morphological changes and affected bacterial survival. The higher temperature resulted in marked changes in the morphology of *C. jejuni* cells, and reduced their culturability and viability in a manner that was dependent on the exposure time. *C. jejuni* has been shown to enter the VBNC state in response to starvation and oxidative stress, and, to a lesser extent, to temperature shifts (3, 14, 16–18, 27). When bacteria were exposed to 48°C, a moderate decrease was observed in *C. jejuni* CFU, which is consistent with the findings of Isohanni *et al.* (13). The altered cell morphology and decline in culturability support the possibility of the transformation of *C. jejuni* into the VBNC form as a consequence of a sublethal heat stress response. In the present study, the VBNC cells were mostly spiral shaped. Thus, we assumed that some VBNC populations may have consisted of both coccoid and spiral VBNC cells (8). It remains unknown whether the VBNC cells induced by the heat treatment maintained their cellular structure and biology, and also whether they retained their virulence with the ability to persist in the environment.

The mechanisms underlying stress regulation need to be elucidated in more detail in order to deepen our understanding of bacterial ecologies, as well as their survival in foods and human hosts. These risks become more apparent as we become more aware of non-culturable cells in potentially dormant forms. Based on the ability of a sub-lethal pre-stress to promote *Campylobacter* resistance, we confirmed the development of acquired resistance by *C. jejuni* cells from the exponential growth phase exposed to 48°C after 5 h starvation.

To survive in a permanently changing world, microorganisms must evolve mechanisms that adjust their biochemistry in response to different signals, and, thus, respond with appropriate alterations in their protein activities that lead to metabolic modifications. Considering the important relationship between physiological cell functions and the proteome of a cell (28), we compared the protein profiles of control and heat-stressed *C. jejuni* cells. The temperature shift to 55°C significantly reduced the number of proteins detected in the gels, corresponding to a reduction in both viability and culturability. Thus, damaged proteins are removed, while *de-novo* synthesis is not possible due to disrupted energy metabolism. As reported by Lazaro *et al.* (21), the small number of proteins detected in the gel in the pH range 4–7 could be attributed to a modification in the isoelectric points of the main protein components, which may result in protein alterations. Down-regulated expression was also detected in *C. jejuni* cells exposed to 48°C, whereas the expression of four proteins was significantly higher than that in the control cells. The latter could be related to the ability of these cells to cope with the sub-lethal heat stress, also resulting in higher viability and culturability (10, 30).

Information on the relationship between stress responses and virulence is limited, especially *in vivo* (14, 27). We previously demonstrated in cell-culture models that heat stress at 55°C strongly impaired bacterial adhesion and invasion in enterocytes (Caco-2, PSI) as well as in macrophages (J774) (31–33). To further define this *in vitro* modulation of the pathogenicity characteristics of *C. jejuni* in response to heat stress, we used an earlier established animal model to provide information on its virulence properties *in vivo*.

The exposure of *C. jejuni* to 48°C or 55°C for 20 min completely abolished its potential to cause systemic infections in a mouse. Despite the morphological changes induced, *C. jejuni* exposed to 55°C for 3 min remained capable of systemic spread; however, the number of bacteria recovered from the livers and spleens was lower than that of the control. These results confirmed that a 3-min exposure to 55°C did not affect the infectivity of *C. jejuni*, but reduced its virulence, which was consistent with our previous findings on starved *Campylobacter* spp. (18, 19).

In contrast, we previously showed that exposure to oxidative stress did not influence the infectivity or virulence of *C. jejuni* because no significant changes were observed in the bacterial load in the mouse liver (19). In the present study, we demonstrated that neither the 3-min nor the 20-min exposure of *C. jejuni* to 48°C affected the course of infection. The number of bacteria recovered from the liver was not significantly reduced 8 d post-infection. The different clearance pattern in the spleen and shorter duration of infection may be explained by different immune mechanisms controlling the infection. The short time exposure to 55°C, as well as to temperatures below this level, appeared to be insufficient to reduce the infectious potential of *C. jejuni*.

New findings relating to the modulation of *C. jejuni* virulence in response to environmental stress factors during food processing are important for providing a better understanding of contamination by and the infective cycle of *C. jejuni*. Elucidating the the different stress response mechanisms in more detail at the cellular level (proteins, metabolism, physiology, virulence) is needed to facilitate the development of appropriate intervention strategies to improve the safety of food supply and reduce the incidence of *C. jejuni*-associated diseases. Regardless of the growth phase, severe heat stress (at 55°C for 20 min) largely eliminated *C. jejuni*, and the adaptive stress response in the fraction of the population that survived did not provide the regulated protein expression that was crucial for their virulence properties *in vivo*. In contrast, the milder sub-lethal heat treatment (at 48°C) allowed for the survival of *C. jejuni* 48°C and its metabolic activity was maintained. The stress responses and cell adaptation induced were visible at the cellular level in terms of their resistance to heat stress as well as metabolic modifications in terms of protein expression. Using the *in vivo* mouse model of campylobacteriosis, we detected differences within the host, concerning the influence of the heat-stress response mechanisms on the reduction of virulence properties, though dependent of the observed organ.
